# *Drosophila* Microbiota Modulates Host Metabolic Gene Expression via IMD/NF-κB Signaling

**DOI:** 10.1371/journal.pone.0094729

**Published:** 2014-04-14

**Authors:** Berra Erkosar Combe, Arnaud Defaye, Noémie Bozonnet, Denis Puthier, Julien Royet, François Leulier

**Affiliations:** 1 Institut de Génomique Fonctionnelle de Lyon, Ecole Normale Supérieure de Lyon, Centre National de la Recherche Scientifique, Université Claude Bernard Lyon-1, Lyon, France; 2 Aix-Marseille Université, Centre National de la Recherche Scientifique, Institut de Biologie du Développement de Marseille-Luminy, Marseille, France; 3 Technological Advances for Genomics and Clinics, Institut National de la Santé et de la Recherche Médicale, Aix-Marseille Université, Marseille, France; University of Bern, Switzerland

## Abstract

Most metazoans engage in mutualistic interactions with their intestinal microbiota. Despite recent progress the molecular mechanisms through which microbiota exerts its beneficial influences on host physiology are still largely uncharacterized. Here we use axenic *Drosophila melanogaster* adults associated with a standardized microbiota composed of a defined set of commensal bacterial strains to study the impact of microbiota association on its host transcriptome. Our results demonstrate that *Drosophila* microbiota has a marked impact on the midgut transcriptome and promotes the expression of genes involved in host digestive functions and primary metabolism. We identify the IMD/Relish signaling pathway as a central regulator of this microbiota-mediated transcriptional response and we reveal a marked transcriptional trade-off between the midgut response to its beneficial microbiota and to bacterial pathogens. Taken together our results indicate that microbiota association potentiates host nutrition and host metabolic state, two key physiological parameters influencing host fitness. Our work paves the way to subsequent mechanistic studies to reveal how these microbiota-dependent transcriptional signatures translate into host physiological benefits.

## Introduction

Metazoans establish functional interactions with their microbiota, the dynamic microbial communities that colonize their mucosal surfaces. These interactions contribute to many aspects of host physiology, notably metabolism and immunity [1]. Despite recent progress, the molecular mechanisms through which the microbiota exerts its beneficial influences on host physiology are still largely undefined.

Recently, *Drosophila melanogaster* has emerged as a powerful model to study host-microbiota interactions [2], [3]. Compared to mammalian species, *Drosophila* carry microbial communities of low complexity, composed of only few dominant bacterial species (mostly of the *Acetobacteraceae* and *Lactobacillaceae* families). The ease to manipulate *Drosophila* commensal bacterial species and to cultivate Germ-Free (GF) animals, coupled to its powerful genetic tools makes *Drosophila* an ideal host model to study molecular mechanisms underlying microbiota-mediated physiological benefits.

*Drosophila* microbiota affects host biology throughout its life cycle [2]–[4]. In adults, *Drosophila* microbiota influences lifespan [5]–[7], shapes mating preference [8], increases host resistance to several intestinal pathogens [9], modulates intestinal immune homeostasis [10]–[12] and promotes intestinal epithelium renewal [13], [14]. During the juvenile (larval) phase the microbiota accelerates animal growth and maturation rate [14]–[16] when the host is under nutritional challenge. These observations point to an important role of *Drosophila* microbiota in shaping the biology of its host. However the molecular dialogue underlying these functional benefits remains elusive.

In this study, we used gnotobiotic *Drosophila* to reveal and study host-microbiota molecular dialogue. To this end, we performed a transcriptome analysis of germ-free and ex-germ-free animals re-associated with a standardized microbiota. Our results demonstrate that microbiota association sustains the expression of genes related to metabolism and digestion in the *Drosophila* midgut, partly via the activity of the IMD/Relish signaling cascade, a pathway previously associated to the regulation of processes related to immune responses. In addition, we further demonstrate that upon bacterial infection in the midgut, the expression of metabolic gene promoted by microbiota association is down-regulated, indicating the existence of host transcriptional trade-off between infection and normal physiology.

## Results and Discussion

### *Drosophila* microbiota impacts midgut genes expression

**Figure 1:**
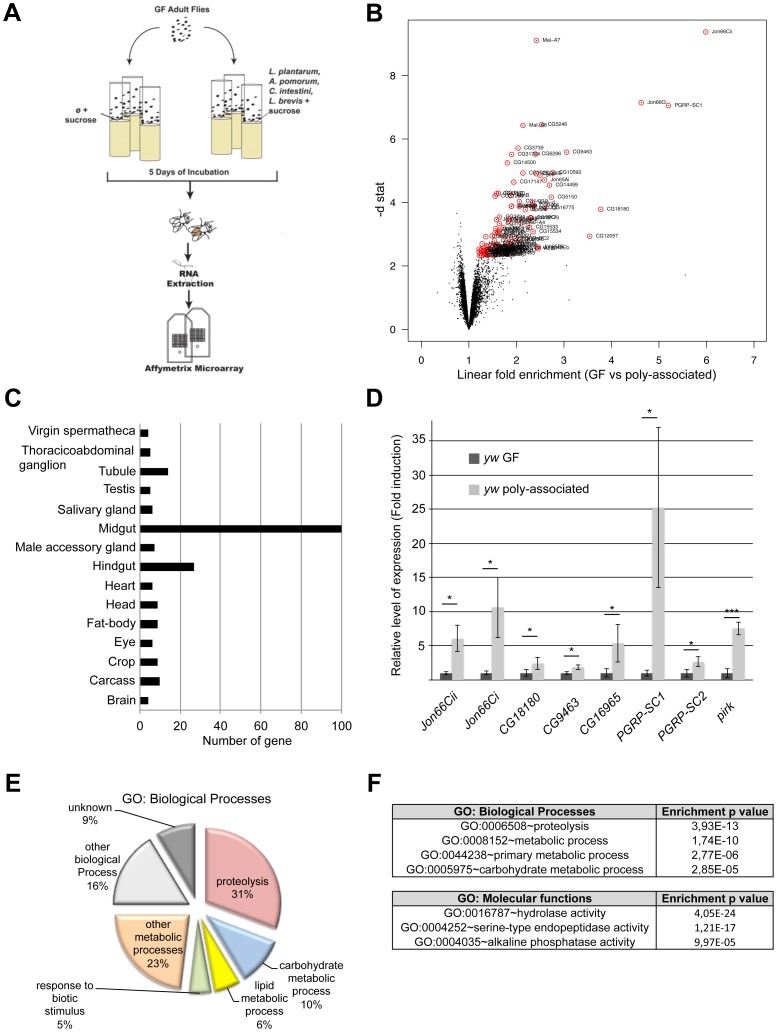
*Drosophila* microbiota impacts midgut genes expression. (A) A schematic representation of the experimental set-up used for transcriptome analysis; GF: Germ-Free. (B) A volcano plot showing the absolute value of the computed statistics from the SAM analysis (d-stat) based on to the fold change in gene expression between the GF and poly-associated groups. The genes selected as differentially expressed by the SAM analysis are highlighted with a red circle around them. The selection was made using a value of Delta that corresponds to a FDR of 0.2. (C) Adult tissue enrichment classification of the 105 microbiota-regulated genes. (D) A bar graph representing the fold-enrichment of different microbiota-regulated transcripts in dissected midguts from GF vs poly-associated wild-type animals (the value of the relative ΔCt*^gene^*/ΔCt*^rp49^* ratio was calculated for every sample and relativized to the ratio in the GF condition which was anchored to 1 to indicate fold induction). Statistical significance of the result is represented (Student's t-test: ns≥0.05>*≥0.01>**≥0.001>***). Of note, *PGRP-SC1* mRNAs are detected to a 16 folds higher level than *PGRP-SC2* mRNA in young poly-associated animals (ΔCt*^PGRP-SC1^*/ΔCt*^rp49^* = 63 and ΔCt*^PGRP-SC2^*/ΔCt*^rp49^* = 4). (E) Relative percentage of Gene Ontology Terms: Biological processes in the list of microbiota-regulated gene (pie chart) and (F) Enrichment p-values (Benjamini-Hochberg corrected) for GO:Biological processes and GO:Molecular Functions in the list of microbiota-regulated genes.

**Figure 2:**
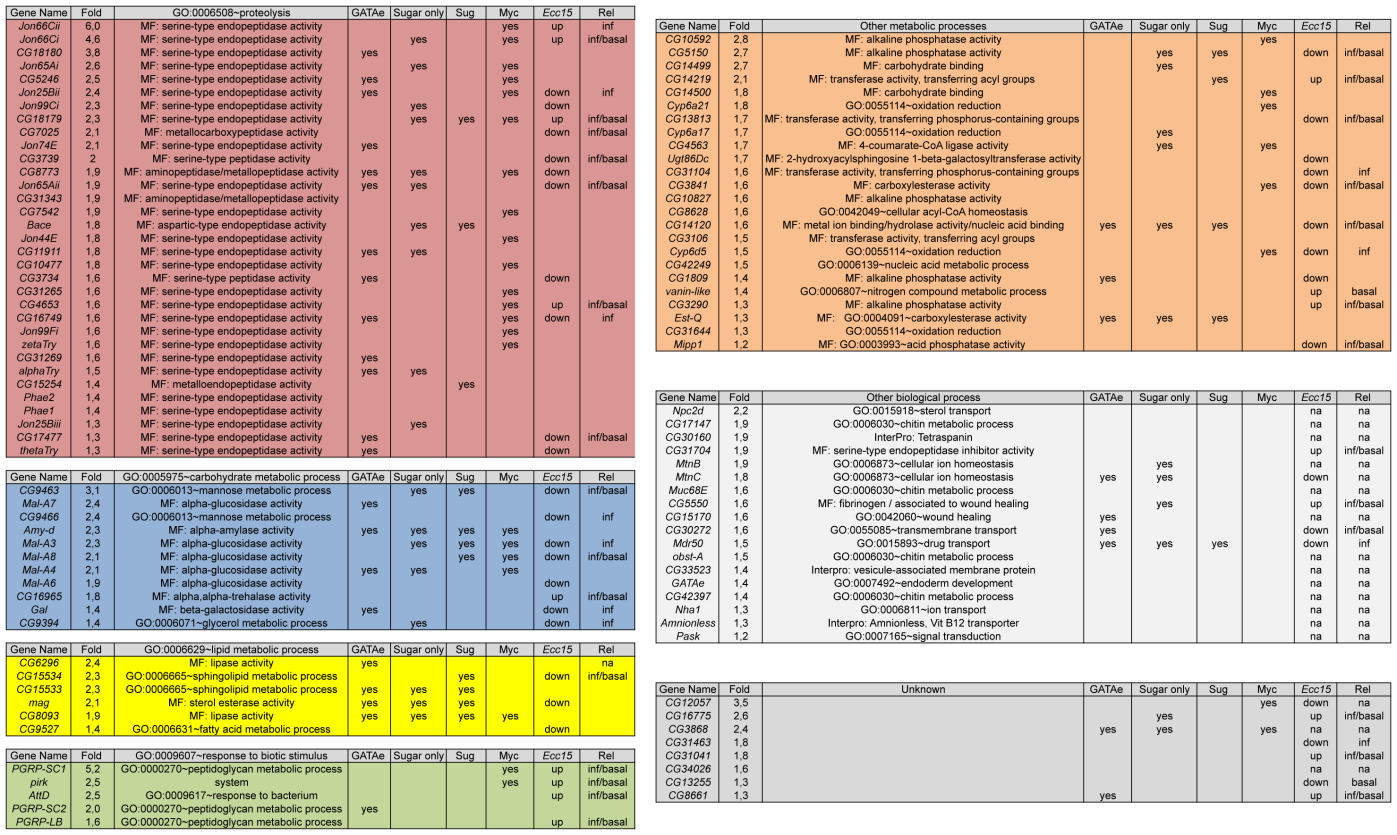
Microbiota-regulated genes. Microbiota-regulated genes are classified by Gene Ontology (GO):Biological function groups. Columns for each microbiota-regulated gene are designated as such: (1) Gene names from Flybase; (2) fold induction in microbiota associated animals vs GF; (3) information on gene function: lower hierarchy GO:Biological process or GO:Molecular function or insight on molecular function by mentioning an Interpro domain present in the protein; (4) Regulation by GATAe; (5) Gene deregulated upon sugar-only diet challenge; (6) Regulation by Sug; (7) Regulation by Myc; (8) Gene regulation in the midgut upon *Ecc15* infection (*Ecc15*) and (9) Relish dependence (Rel regulation) for gene expression in the midgut in basal condition (basal) or upon *Ecc15* infection (inf). For columns “GATAe” the data were collected from [21], [27]; columns “Sugar only” and “Sug” data are from [29]; column “Myc” data are from [28]; columns “*Ecc15*” and “Rel” data are from [31].

To gain insight into the molecular cross-talk between *Drosophila* microbiota and its host, we compared the transcriptomic changes between microbiota-associated adult flies and their Germ-Free (GF) siblings. Since the microbiota load and composition encountered in conventionally laboratory-reared flies (CONV) fluctuate highly [9], [17] (and our unpublished observation), we chose to associate newly emerged GF adults with a standardized microbiota composed of four previously characterized *Drosophila* commensal bacterial strains (*Acetobacter pomorum*, *Commensalibacter intestini, Lactobacillus brevis* and *Lactobacillus plantarum*) [18] (Fig.1A). After data normalization and statistical analysis using Significance Analysis of Microarrays (SAM), using a false detection rate (FDR) of 0,2, we identified 105 transcripts whose expression level were significantly increased from 1.2 to 6 folds in the polyassociated flies compared to their GF siblings (Fig.1B and Fig.2).

We classified the 105 selected genes according to their annotated tissue expression profile using the FlyMine tool [19] based on the FlyAtlas dataset [20]. Interestingly, despite the fact that the transcriptomic analysis was conducted on whole adult animals, we found that most (100/105) of the selected transcripts are expressed in the midgut of conventionally (CONV) raised adults (i.e with a microbiota) (Fig.1C). Recently, Buchon *et al.* and Marianes *et al.* reported that 60–65% of the *Drosophila* genes are detected in the midgut of CONV adults with specific patterns of expression in this tissue [21], [22]. We therefore examined whether our list contains enrichment for genes sharing a given spatial pattern of expression in the midgut but we failed to detect any (data not shown). Nonetheless, the marked over-representation of midgut genes in our dataset indicates that the host transcriptional response to the microbiota is localized and occurs mainly in the *Drosophila* midgut. We then verified that microbiota-regulated transcripts were indeed enriched in the midgut upon microbiota-association. To do so, using RT-qPCR, we compared the expression levels of a set of the most strongly microbiota-regulated genes in dissected midguts from GF and microbiota-associated animals and found that the tested genes were all up-regulated in adult midguts associated with four commensal bacterial strains compared to GF controls (Fig.1D). These results therefore demonstrate that microbiota association impacts on genes expression in the midgut.

### *Drosophila* microbiota sustains metabolic genes expression

We next used Gene Ontology (GO) clustering and enrichment analysis tools (Database for Annotation, Visualization, and Integrated Discovery or DAVID; [23]) to identify the functional categories within our gene list. We found that 74 out of 105 genes on the list are associated with metabolic processes (Fig.1E, Fig.2). Among the 74 genes, 33 are involved in proteolysis, 11 in carbohydrate metabolic process, six in lipid metabolic process and four in oxydo-reduction. From the same analysis, we found that the terms “metabolic process”, “proteolysis”, “primary metabolism” and “carbohydrate metabolism” are the most significantly enriched terms (Fig.1E–F, Fig.2). Among the remaining 31 genes, several are involved in processes that could either be connected to metabolic activities such as transport across membranes (4/31), chitin metabolism (4/31) and cellular ion homeostasis (2/31) or linked to host response to bacterial colonization (i.e, “response to biotic stimulus” (5/31) and “wound healing and related process” (2/31)) (Fig.1E and Fig.2). These results not only indicate that *Drosophila* presents an expected transcriptional response to bacterial colonization upon microbiota association, but also reveal a clear impact of microbiota association on host metabolic processes at least at the transcriptomic level.

Using the same GO terms tool, we further analyzed the molecular functions associated with the 105 genes (Fig.2). We identified a clear enrichment of genes encoding hydrolases and alkaline phosphatases (Fig.1F), which are two major classes of enzymes involved in digestive functions, suggesting that *Drosophila* microbiota enhances the host's enzymatic activities for macronutrients breakdown. Strikingly, among the 74 “metabolic genes”, 60 genes encode hydrolases, including 33 peptidases (28 serine-proteases including several members of the *trypsin* and *Jonah* proteases families, four metallo-proteases and the aspartic-protease *Bace*), 10 glycosyl-hydrolases (five alpha-glucosidases of the *maltase* family, two alpha-mannosidases, the alpha-amylase *Amy-d*, the beta-galactosidase *Gal* and one alpha,alpha-trehalase), six phosphatases (five alkaline and the acid-phosphatase *Mipp1*), five lipases (including *magro*, the *Drosophila* LipA homologue controlling cholesterol homeostasis, [24]), three non-lipase esterases (including *EstQ*), two nucleases and the biotinidase *vanin-like* (Fig.2). The other metabolic genes include five transferases (three acyl-transferases, one phospho-transferase and the beta-galactosyl-transferase *Ugt86Dc*), three Cytochrome P450 (*Cyp6a17*, *Cyp6a21* and *Cyp6d5*), two Carbohydrate binding proteins, two genes related to Acyl-CoA metabolism, one Cytochrome-c oxidase and one 4-Coumarate-CoA ligase. These molecules are mostly involved in primary metabolic pathways, suggesting that microbiota association sustains these essential host metabolic activities. This finding is consistent with the observation that microbiota influences host energy homeostasis and carbohydrate allocation patterns in adults [15]. Among the 31 “non metabolic genes” regulated by microbiota association, we identified 11 genes that are still related to metabolic activities, which may be influenced by microbiota association. Five out of the 11 genes are involved in transport of micronutrients or xenobiotics including the vitamin-B12 transporter *Amnionless*, the sterol transporter *Npc2d* and the sodium transporter *Nha1*; four genes are associated to chitin metabolism, including the mucin *Muc68E* and the chitin binding molecule *obst-A* and two genes encode proteins involved in metal homeostasis (*MtnB/C*). In addition to these metabolic signatures, we identified seven genes that are clearly associated to host tissue response to bacterial challenges. Specifically, three of the seven genes are associated to “wound healing”, such as one fibrinogen; five are innate immune genes whose expression is known to be controlled by the IMD signaling pathway such as the PGRP-LC/LE inhibitor *pirk* and the peptidoglycan amidases *PGRP-LB, -SC1* and *-SC2* which are all involved in dampening the IMD signaling strength to promote immune tolerance to indigenous microbiota [10]–[12]. This observation corroborates previous reports demonstrating that the gut microbiota modulates intestinal immune homeostasis and promotes intestinal epithelium renewal [4]. Finally, we identified the Zinc-finger transcription factor GATAe, which is required for the terminal differentiation of the *Drosophila* endoderm and maturation of the adult midgut [21], [25], [26]. Interestingly, among the 105 genes uncovered by our transcriptomic analysis, we could identify 31 genes which expression is altered upon *GATAe* genetic manipulation [21], [27] (Fig.2). This observation reinforces the notion that microbiota may promote the maturation and the digestive functionalities of the midgut partly via GATAe-dependent regulation of digestive enzymes expression.

Taken together, our results clearly indicate that microbiota association influences the expression of host midgut genes encoding key actors involved in digestive functions, primary metabolism and host tolerance to bacteria colonization and that *Drosophila* microbiota sustains these activities.

### Correlation between microbiota and nutrients-mediated transcriptional signatures

Metabolic adaptation through metabolic gene regulation is essential for the host to respond to nutritional challenges. Now, having observed that microbiota association promotes the transcription of metabolic genes, we further compared our results with previous analysis on *Drosophila* transcriptome upon nutritional challenges [28], [29]. Among the 105 microbiota-regulated genes, the expression of 30 genes was reported to fluctuate in response to sugar only diet [29] (Fig.2). Specifically, Zinke *et al.* reported that *sugarbabe (sug)*, a zinc-finger transcription factor that is strongly activated upon sugar ingestion, represses the expression of several genes involved in dietary sugar and fat breakdown. We found in our list 16 “*sug*-regulated” genes among which four are Glycosyl-hydrolases (*Amy-d, CG9463, Mal-A3* and *Mal-A8*) and four are lipases (*CG15534*, *CG8093*, *mag* and *CG15533*). In our experimental conditions, flies were reared on a sucrose-only diet prior and during the association. Therefore, the upregulation of *sug*-related genes upon microbiota association suggests that the repressive activity of Sug during sugar feeding is inhibited during host response to microbiota. Similarly, Li *et al.* identified the transcription factor Myc as one of the main regulators of metabolic genes expression in response to nutritional challenges. In this study we found 30 Myc-regulated genes in our list [28] (Fig.2). This correlation suggests that Myc is also a prime candidate to mediate the transcriptional host response to microbiota association.

In summary, the host transcriptomic response to microbiota association includes the modulation of a significant number of genes required to adapt to nutritive challenges. This finding suggests that microbiota association potentiates nutrition via enhanced digestive enzyme expression mediated at least partly via Sug inhibition and Myc activation.

### Trade-off between microbiota-mediated and infection-mediated midgut genes expression

**Figure 3:**
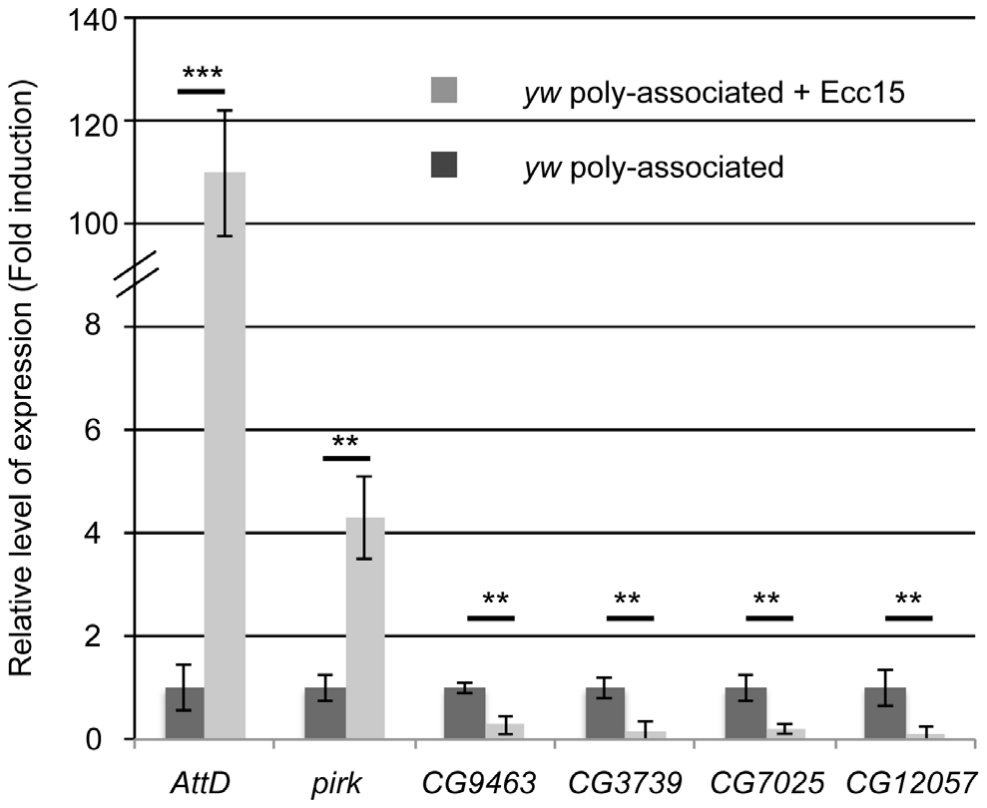
*Ecc15* infection promotes expression of microbiota-regulated immune genes while repressing microbiota-regulated metabolic genes in midguts. A bar graph representing the fold-enrichment of different microbiota-regulated transcripts in dissected midguts from poly-associated upon oral infection by *Ecc15* in WT animals (the value of the relative ΔCt*^gene^*/ΔCt*^rp49^* ratios was calculated for every sample and relativized to the ratio in the GF condition which was anchored to 1 to indicate fold induction). Statistical significance of the result is represented (Student's t-test: ns≥0.05>*≥0.01>**≥0.001>***).

Buchon *et al.* characterized the transcriptional signatures in dissected CONV adult midguts after an acute oral infection with the bacterial strain *Erwinia carotovora carotovora 15* (*Ecc15*). Our current study shares many common features with that of Buchon *et al.*, in that we adopted similar experimental protocols to study changes in transcriptomes after exposing the host to a specific set of bacteria and we both found a marked transcriptional response localized to the midgut. We therefore compared our dataset to that of Buchon *et al*. and found an evident overlap between the two lists: half of our microbiota-regulated genes are also modulated upon intestinal infection (52/105 genes, Fig.2). As expected, like in Buchon *et al.*, we also found that the IMD pathway target genes (*AttD, pirk, PGRP-LB/-SC1/-SC2*) were up-regulated. However, most microbiota up-regulated genes (35/52) were in fact down-regulated upon *Ecc15* infection, suggesting the existence of a transcriptional trade-off between the response to indigenous bacteria (i.e the microbiota) and the response to infectious bacteria. We tested this hypothesis by infecting the flies associated with a standardized microbiota with *Ecc15* and studying the expression of a selection of candidate genes from our list (Fig.3). As expected, we found that the immune-related genes *pirk* and *AttD* are up-regulated, and we confirmed that several microbiota-regulated genes such as digestive enzymes (*CG3739*, *CG7025*, *CG9463*), are markedly down-regulated upon *Ecc15* infection. This result reinforces the notion that *Ecc15* infection triggers a transcriptional trade-off to promote immune-related genes expression at the expense of the metabolic genes expression up-regulated by the microbiota association.

### IMD/Relish pathway at least partly controls the expression of microbiota-regulated metabolic genes

**Figure 4:**
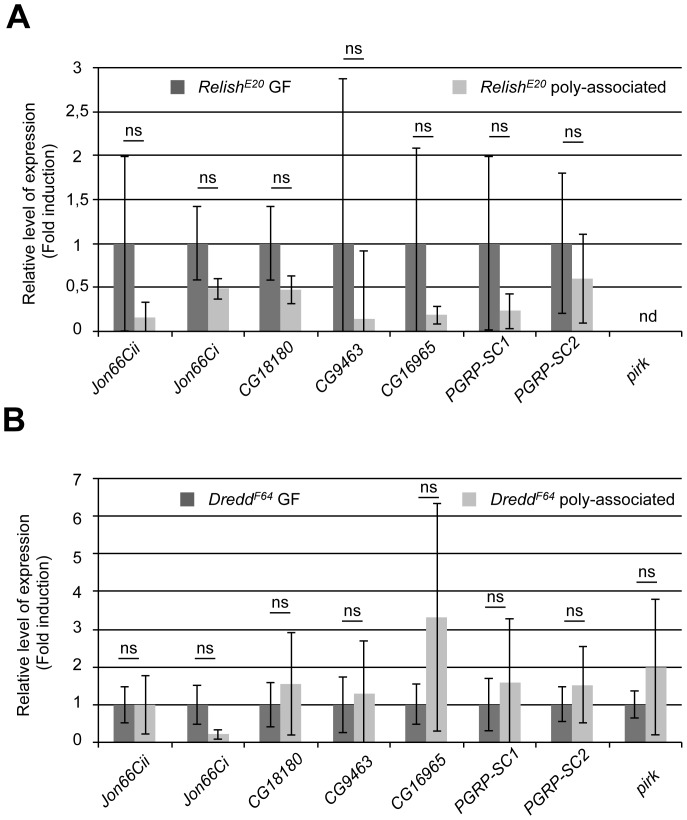
IMD/Relish signaling controls microbiota mediated transcript enrichment in midguts. Bar graphs representing the fold enrichment of different microbiota-regulated transcripts in dissected midguts from GF vs poly-associated *Relish^E20^* (A) or *Dredd^F64^* (B) mutant animals (the values of the relative ΔCt*^gene^*/ΔCt*^rp49^* ratios were calculated for every sample and relativized to the ratio in the GF condition which was anchored to 1 to indicate fold induction). Statistical significance of the result is represented (Student's t-test: ns≥0.05>*≥0.01>**≥0.001>***). nd: not detected.

*Relish* encodes the *Drosophila* orthologue of the mammalian NF-kappaB factor p105, which functions downstream of the IMD signaling pathway and controls the induction of most IMD target genes [30]. Buchon *et al.* studied the midgut transcriptome of *Relish* mutants and found that changes in the expression of midgut genes upon *Ecc15* infection are largely *Relish*-dependent [31]. Strikingly, from our list of genes, the vast majority of the 52 microbiota-regulated genes influenced by *Ecc15* infection are also Relish-dependent (41/52; Fig.2). Based on the dataset from Buchon *et al.*, we derived the information of *Relish* dependence for the basal expression level of each midgut gene in CONV animals in their study, and compared this dataset with our list of microbiota-regulated genes. We found that 39 of the microbiota-regulated genes rely on Relish activity for their basal expression in the midgut of CONV animals (Fig.2). Strikingly, all these 39 genes were also regulated in a *Relish* dependent manner upon *Ecc15* infection in CONV animals. These observations suggest that Relish, in addition to its known role to control the expression of immune-related genes, may also be an important transcriptional regulator of metabolic genes induced by the microbiota, which are likely independent of immune responses. To test this hypothesis, we studied the expression of a set of eight microbiota-regulated genes in the midguts of two mutants of the IMD signaling pathway: *Dredd* and *Relish*. *Dredd* encodes the *Drosophila* orthologue of Caspase-8 whose function is essential for IMD pathway signal transduction and Relish activation [32]. In *Dredd* or *Relish* mutant background, both immune-related (*pirk, PGRP-SC1/SC2*) and metabolism-related (*Jon66Cii, Jon66Ci, CG16965, CG18180* and *CG9463*) microbiota-regulated genes are no longer induced in the midgut upon standardized microbiota association (Fig.4). These results demonstrate that microbiota impacts metabolic gene expression partly via IMD/Relish activity.

### Conclusion

The *Drosophila* indigenous microbiota modulates host physiology [2], [3]. In this study, we have identified molecular signatures associated to these effects and pinpointed the central role of the IMD/Relish signaling pathway in controlling host transcriptional response to its microbiota. One striking result in our study is that the host transcriptome response to microbiota association is mostly restricted to the midgut, a major biological interface between the host and its environment and the primary site where host/microbiota interactions occurs [4]. As described in previous studies, microbiota association triggers a transcriptional change related to host response to bacteria with similar molecular signatures (i.e, immune-related genes) to those elicited by pathogenic bacteria infection. However, microbiota association clearly favors a unique transcriptional response funneled towards promoting host metabolic capacities. Such response is severely dampened upon bacterial infection as a trade-off for the host to mount potent immune and tissue repair responses. Since the IMD/Relish pathway is instrumental to promote both the metabolic response to microbiota association and the response to infection, it is likely that the transcription factor Relish is at the cornerstone of the transcriptional trade-off between the midgut response to beneficial microbiota and response to midgut pathogens. Other factors may also contribute to this trade-off, such as ATF3, which was recently reported to control immune and metabolic homeostasis in the *Drosophila* midgut [33].

Taken together, our results demonstrate that *Drosophila* microbiota has a marked impact on the expression of genes mainly involved in digestive functions and primary metabolism, suggesting that microbiota association potentiates host nutrition and host metabolic state, two key physiological parameters contributing to host fitness. Our results are in agreement with recent reports demonstrating that microbiota influence adult nutritional and metabolic phenotypes [15] and therefore pave the way to the subsequent mechanistic studies on how these microbiota-dependent transcriptional responses translate into host physiological benefits.

## Materials and Methods

### *Drosophila* lines and breeding

*Drosophila* were cultured at 25°C on a standard yeast/cornmeal medium containing 10 g.L^−1^ agar (VWR, ref. #20768.361), 80 g.L^−1^ cornmeal flour (Westhove, Farigel maize H1), 50 g.L^−1^ inactivated dry yeast (Bio Springer, Springaline BA95/0-PW), 5.2 g.L^−1^ Methylparaben sodium salt (MERCK, ref. #106756) and 4 ml.L^−1^ of 99% propionic acid (CARLO ERBA, cref. #409553). Germ-free animals were obtained from bleached embryos cultured on autoclaved conventional medium. When needed GF stocks were maintained during few generations on antibiotic supplemented food (final concentration: 50 µg.L^−1^ ampicilline, 50 µg.L^−1^ kanamicine, 15 µg.L^−1^ erythromycin, 50 µg.L^−1^ tetracycline) to avoid bacterial contamination. *Drosophila y,w* flies were used as the reference strain in this work. The following mutant lines were used: *y,w,Dredd^F64^* and *y,w;;Relish^E20^* [34].

### Bacterial strains and culture conditions

Erwinia carotovora carotovora^15^ (Ecc15), Lactobacillus plantarum^WJL^, Lactobacillus brevis^EW^, Commensalibacter intestini^A911T^, and Acetobacter pomorum strains were used in this study. All strains were described previously [18], [35]. A. pomorum and C. intestini were cultivated in Mannitol medium (3 g.L^−1^ Bactopeptone (Difco, cat. #0118-17), 5 g.L^−1^ yeast extract (Difco, cat. #212750), 25 g.L^−1^ D-Mannitol (Sigma, ref. M1902) at 30°C at least for 18 hours on agitation, L. brevis and L.plantarum were cultivated in Man, Rogosa and Sharpe (MRS) broth (Difco, ref. #288110) at 37°C standing. Ecc15 was cultivated in Luria-Bertani broth medium (Difco, ref. #244610) at 30°C for 24 hours on agitation.

### Poly-association of GF adults with a standardized microbiota and Ecc15 infection

Association mixture: 150 µL of bacterial solution made of 75 µL of 5% sucrose solution sterilized by filtration through a 0.2 µm membrane (Pall Life Sciences, ref. #4652)+75 µL of a mix of equal amounts of four commensal bacterial cultures (*Lactobacillus plantarum, Lactobacillus brevis, Commensalibacter intestini* and *Acetobacter pomorum*) at an initial OD_600_ = 1.0 each. Control mixture: 150 µL of a control solution (75 µL filter-sterilised sucrose +75 µL of a 1∶1 mixture of sterile MRS and sterile Mannitol). Either of these solutions was added to a disc of autoclaved paper (Whatman, ref. #3030917) covering standard fly culture media in standard fly tubes. For *Ecc15* infection of poly-associated flies, *Ecc15* culture at final OD_600_ = 100 resuspended in PBS was added to the mix of commensal bacterial strains and inoculated onto autoclaved paper disc. Germ-free adults derived from freshly made GF embryos were collected within the first 48 hours of adult emergence and placed in groups of 25–30 females and 10 males in experimental tubes. Flies were kept in such tubes for 2 days at 25°C and transferred into newly prepared vials with fresh bacterial cultures. 3 days after transfer, on day 5 post-inoculation flies were either sampled or infected with *Ecc15* for 8 hrs. In each experiment we measured internal bacterial loads of representative experimental animals by plating fly homogenates on MRS and Mannitol agar plates to check the effectiveness of the re-association and infection processes or the axenic status of the flies. On average flies carried 10^4^ CFUs/animal upon re-association and >10^6^ CFUs/animals upon infection.

### Microarray analysis

Three biological replicates of 20 females for each condition (GF or poly-associated) were homogenized in 500 µl of Trizol (Invitrogen) and 100 µl Choloroform using 0.75–1 mm glass beads and the Precellys24 Tissue Homogenizer (Bertin Technologies, France). RNA pools were isolated and purified using NucleoSpin RNA kit (Macherey-Nagel). RNA was quantified on NanoDrop ND-1000 and RNA quality was controlled on Agilent 2100 Bioanalyzer chips. For each sample, 1 µg of total RNA was amplified and labeled using the GeneChip IVT Labeling Kit according to the protocol provided by the supplier. Affymetrix GeneChip *Drosophila* Genome 2.0 arrays were hybridized with 7.5 µg of labeled cRNA, washed, stained and scanned according to the Affymetrix' protocols. Raw data are deposited at NCBI GEO with the accession number: GSE56173. Raw data analysis was performed using R for Statistical Computing (R core team 2013) with Robust Multi-array Average (RMA) and Significance Analysis of Microarrays (SAM) from the package *siggenes* (http://bioconductor.wustl.edu/bioc/html/siggenes.html). Gene selection was made using a value of Delta that correspond to a FDR of 0.2. Gene Ontology Term analysis and enrichments were made using DAVID [23].

### RNA extraction and RT-qPCR analysis

Three biological replicates of a pool of 10 midguts dissected from females were used for RT-qPCR analysis. Foregut, hindgut, crop and malpighian tubes were carefully removed. Tissue homogenization, RNA extraction and purification were performed as described above for whole flies. Reverse transcription of 300 ng of RNA was performed using Superscript II enzyme (Invitrogen) and random primers (Invitrogen). Quantitative PCR was performed on a Biorad CFX96 apparatus (Biorad) using SYBR GreenER qPCR Supermix (Invitrogen), cDNA (1/160 dilution of the reverse transcription products) and gene specific primer sets (available upon request). Melting curves of the detected amplicons were analysed to ensure specific and unique amplification. PCR efficiency was calculated using serial dilution of cDNA. We used the ΔΔCt method for data analysis and *rp49* as the reference gene. Results were expressed as a relative value of ΔCt*^gene^/*ΔCt*^rp49^* ratios (fold induction).
