# Correction: *Drosophila* Microbiota Modulates Host Metabolic Gene Expression via IMD/NF-κB Signaling

**DOI:** 10.1371/journal.pone.0104120

**Published:** 2014-07-25

**Authors:** The PLOS ONE Staff

The abbreviation of the first author’s name is incorrect in the citation. The correct abbreviation is: Erkosar B. The correct citation is: Erkosar B, Defaye A, Bozonnet N, Puthier D, Royet J, et al. (2014)*Drosophila* Microbiota Modulates Host Metabolic Gene Expression via IMD/NF-κB Signaling. PLoS ONE 9(4): e94729. doi:10.1371/journal.pone.0094729
